# A novel scoring system for the quantitative prediction of prognosis in acute myeloid leukemia

**DOI:** 10.3389/fonc.2023.1144403

**Published:** 2023-03-30

**Authors:** Yang Yu, Hao Wang, Jing-Jing Yang, Shu Fang, Ya-Nan Wen, Yi-Fan Jiao, Kun Qian, Ning Le, Ruo-Qi Shan, Wen-Jing Gao, Bao-Lai Hua, Fei Li

**Affiliations:** ^1^ Department of Hematology, Beijing Shijitan Hospital, Capital Medical University, Beijing, China; ^2^ Department of Hematology, Peking University Ninth School of Clinical Medicine, Beijing, China; ^3^ Department of Hematology, The Fifth Medical Center of Chinese PLA General Hospital, Beijing, China; ^4^ Medical School of Chinese PLA, Beijing, China; ^5^ School of Medicine, Nankai University, Tianjin, China; ^6^ The Second School of Clinical Medicine, Southern Medical University, Guangzhou, China

**Keywords:** AML, prognostic model, prognosis prediction, risk scoring model, risk stratification

## Abstract

**Background:**

Acute myeloid leukemia (AML) is a heterogeneous hematopoietic malignancy. Patient prognosis cannot be accurately assessed in National Comprehensive Cancer Network (NCCN) risk stratification subgroups based on the current criteria. This study aimed to develop a novel prognostic score model for the quantitative prediction of prognosis in AML.

**Results:**

We developed a prognostic risk scoring model of AML using differentially expressed genes to predict prognosis in patients with AML. Furthermore, we evaluated the effectiveness and clinical significance of this prognostic model in 4 AML cohorts and 905 patients with AML. A prognostic risk scoring model of AML containing eight prognosis-related genes was constructed using a multivariate Cox regression model. The model had a higher predictive value for the prognosis of AML in the training and validation sets. In addition, patients with lower scores had significantly better overall survival (OS) and even-free survival (EFS) than those with higher scores among patients with intermediate-risk AML according to the NCCN guidelines, indicating that the model could be used to further predict the prognosis of the intermediate-risk AML populations. Similarly, patients with high scores had remarkably poor OS and EFS in the normal-karyotype populations, indicating that the scoring model had an excellent predictive performance for patients with AML having normal karyotype.

**Conclusions:**

Our study provided an individualized prognostic risk score model that could predict the prognosis of patients with AML.

## Background

Acute myeloid leukemia (AML) is a heterogeneous hematopoietic malignancy characterized by malignant clonal proliferation of stem cells, with complex pathogenesis and widely varying clinical prognosis. At present, several prognostic systems have been established, such as the European Leukemia Net system and National Comprehensive Cancer Network (NCCN) guidelines. These standards are widely accepted and applied by clinicians, and are mainly based on chromosomal variants and gene mutations. The clinical outcomes are prominently heterogeneous in cytogenetically normal AML (CN-AML) (1), which accounts for about half of all adult patients with AML having an intermediate prognosis (2). Although several other molecular markers, such as FLT3, NPM1, and CEBPA mutations, have been clinically validated and have greatly improved the prognostic classification of AML (3), the patients’ risk cannot be further accurately assessed in NCCN risk stratification subgroups based on current criteria. Hence, recognizing that the current risk evaluation systems cannot accurately represent the clinical risk of each patient, especially for patients with intermediate risk of NCCN, is important.

In recent years, numerous aberrant-expression genes have been presented as the prognostic factors for AML, such as *ERB, DNMT3B, BAALC*, and so on. High *DNMT3B* expression is independently associated with adverse outcomes in older patients with CN-AML (4). *CASP1* is highly expressed in AML cells, especially in relapsed leukemic cells. High *CASP1* expression was associated with poor prognosis, and *CASP1* inhibition could impair the proliferation of AML cells (5). The presence of numerous abnormal expression genes due to chromatin abnormalities or mutations is closely related to AML prognosis. Part of these genes are expressed only in patients with AML or differentially expressed in patients and healthy individuals. Therefore, we should focus more on studying differentially expressed genes (DEGs) when studying the factors affecting AML prognosis. It is difficult to accurately assess the clinical risk of patients, and hence AML remains one of the most challenging disorders to treat (6). Therefore, it is particularly important to accurately stratify patients with AML, so as to choose preferred treatment regimens and improve the prognosis of patients. This study aimed to develop a novel risk score model for the quantitative prediction of prognostic risk in AML based on aberrantly expressed genes in patients with AML.

## Results

### A total of 354 genes were differentially expressed in patients with AML and healthy individuals, and 8 of these genes were screened out to construct a prognostic score model

**Figure 1:**
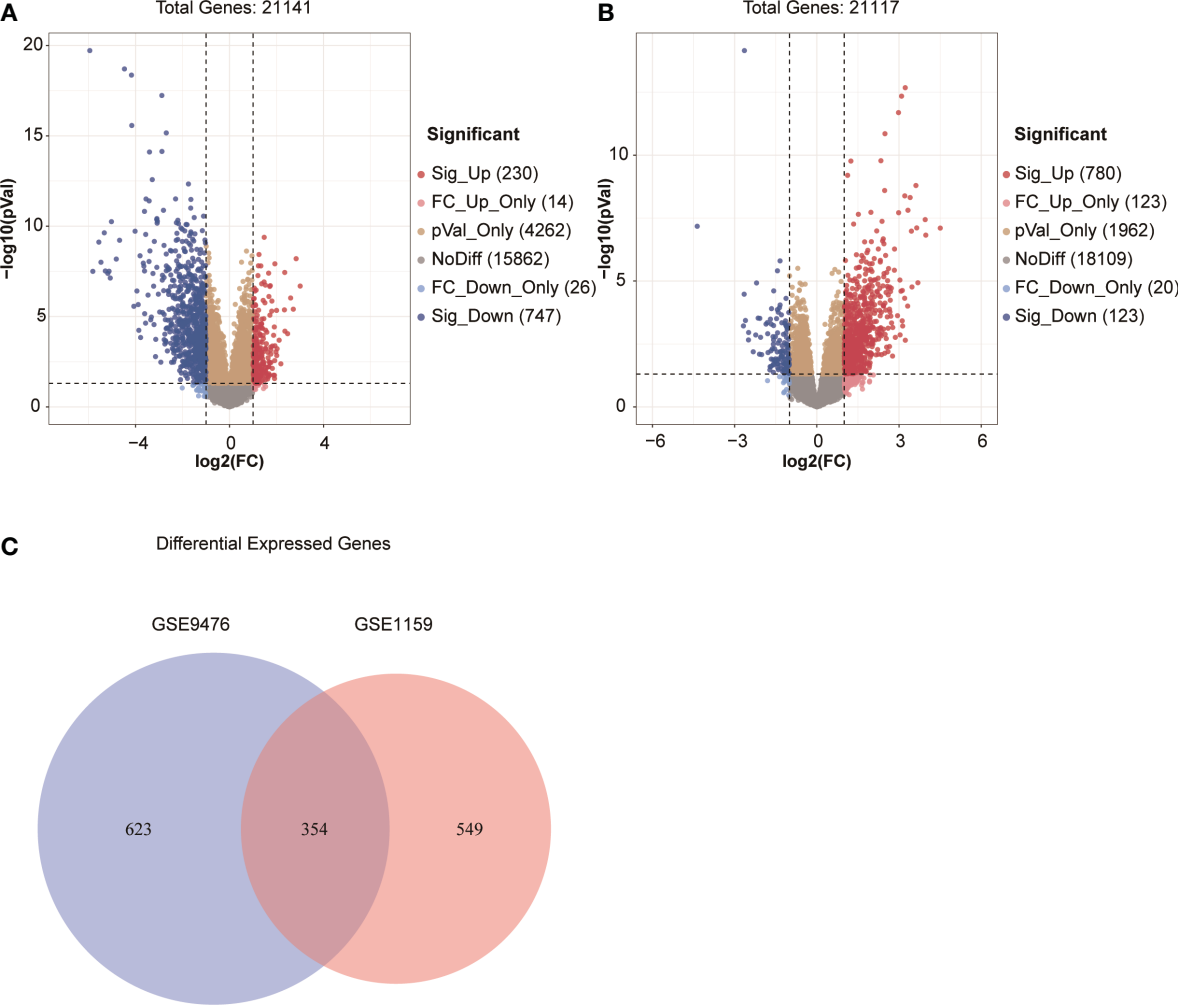
**(A, B)** Volcano map of DEGs in GSE9476 and GSE1159. **(C)** Venn diagram of DEGs from the two databases.

We screened DEGs (adj.*P <*0.05 and |log2FC| >1) between patients with AML and healthy individuals in two independent microarray expression profiles GSE9476 and GSE1159 to identify the DEGs of patients with AML compared with healthy individuals. Further, 977 significant DEGs were found in GSE9476, of which 230 genes were significantly upregulated and 747 were significantly downregulated in patients with AML (**Figure 1A**). In GSE1159, 903 DEGs were detected in patients with AML and healthy individuals, of which 780 genes were significantly upregulated and 123 genes were significantly downregulated in patients with AML (**Figure 1B**). Taking the intersection of the DEGs in the 2 databases, 354 DEGs was obtained in both databases (**Figure 1C**). The 354 DEGs shared in the GSE9476 and GSE1159 data sets were analysed using a univariate Cox regression model, and 74 genes related to prognosis were obtained (**Table S1**). A multivariate Cox regression analysis was performed on the 74 genes to obtain an AML prognostic risk score model composed of 8 genes: Risk score = CD58(–0.2484) + CLIC3 × 0.3613 – CUX1 × 0.7672 + ECE1 × 0.1624 + ETS2 × 0.4580 + SFXN3 × 0.2551 + SLC25A12 × 0.6498 + SQLE × 0.1993.

### Clinical and molecular characteristics of patients with AML having a high-risk score

The prognostic score of each patient was calculated using the formula to reveal the clinical and molecular characteristics of patients with AML having different prognostic scores. The 163 patients with AML in the TCGA data set were divided into a high–prognostic score group and a low–prognostic score group (**Table 1**) based on the median prognostic score as the cutoff value. Additionally, corresponding clinical features of those samples that were significantly associated with prognosis were analyzed using Pearson´s correlation test or Fisher’s precision probability test. Compared with the low-score group, patients in the high-score group were older (*P* < 0.001). No significant difference was found in sex, WBC count, ratio of bone marrow blasts, and FAB classification between the two groups. Also, no significant difference was observed in chemotherapy and transplantation regimens between the two groups (**Table 1**). The comparison of mutant and fusion genes in the two groups showed that the occurrence rate of *CBFB–MYH11, RUNX1–RUNX1T1*, and *INSR* increased significantly in the low–prognostic score group (*P* = 0.005, *P* = 0.007, *P* < 0.001) and the occurrence rate of TP53 increased in the high–prognostic score group (*P* < 0.001, **Table 1**). Moreover, in the high–prognostic score group, the genes *DNMT3B* (*P* < 0.001), ITGA3 (*P* < 0.001), and *TCF4* (*P* < 0.001) tended to be highly expressed (**Table 1**). The risk stratification was performed as per the NCCN guidelines for the 163 samples. The number of patients with poor prognosis in NCCN stratification in the high-score group was far more than that in the low-score group (*P* < 0.001). Also, the number of patients with good prognosis in NCCN stratification in the high-score group was significantly less than that in the low-score group (*P* < 0.001, **Table 1**).

**Table 1:** Clinical and molecular characteristics of patients with AML having high and low risk scores.

Variable	*Risk* ^Low^ (*n* = 82)	*Risk* ^High^ (*n* = 81)	*P* value
Sex, female, no. (%)	42 (51.2)	45 (55.6)	0.579
Age, median, year (range)	55 (18–81)	63 (21–88)	<0.001
WBC count, median ×10^9^/L (range)	19.3 (0.6–223.8)	22.2 (0.7–297.4)	0.424
BM blasts, median % (range)	71 (30–100)	73 (30–99)	0.578
PB blasts, median % (range)	42 (0–97)	39 (0–98)	0.337
FAB subtype, no. (%)			
M0	3 (3.7)	13 (16.3)	0.009
M1	21 (25.9)	23 (28.7)	
M2	24 (29.6)	16 (20.0)	
M4	23 (28.4)	12 (15.0)	
M5	10 (12.3)	11 (13.8)	
M6	0 (0)	2 (2.5)	
M7	0 (0)	3 (3.8)	
Fusion gene, no. (%)			
*CBFB–MYH11*	11 (13.4)	1 (1.2)	0.005
*RUNX1–RUNX1T1*	7 (8.5)	0 (0)	0.007
*BCR–ABL1*	0 (0)	3 (3.7)	0.120
Mutated gene, no. (%)			
*FLT3*-ITD	12 (14.6)	20 (14.7)	0.106
*FLT3*-TKD	7 (8.5)	5 (6.2)	0.766
*NPM1*	22 (26.8)	26 (32.1)	0.461
*NPM1* ^Mut^/*FLT3*-ITD^WT^	17 (20.7)	12 (14.8)	0.323
Biallelic *CEBPA*	7 (8.5)	1 (1.2)	0.064
*IDH1*	9 (11.0)	7 (8.6)	0.617
*IDH2*	10 (12.2)	7 (8.6)	0.458
*RUNX1*	4 (4.9)	13 (16)	0.018
*DNMT3A*	20 (24.2)	23 (28.4)	0.562
*TP53*	0 (0)	15 (18.5)	<0.001
*KIT*	6 (7.3)	1 (1.2)	0.117
*ASXL1*	0 (0.0)	3 (3.7)	0.120
*TET2*	11 (13.4)	5 (6.2)	0.098
*NRAS*	8 (9.8)	5 (6.2)	0.565
*WT1*	6 (7.3)	4 (4.9)	0.746
*PTPN11*	3 (3.7)	5 (6.2)	0.495
*KRAS*	3 (3.7)	4 (4.9)	0.720
*MT-CO2*	4 (4.9)	4 (4.9)	1.000
*TTN*	2 (2.4)	4 (4.9)	0.443
*U2AF1*	2 (2.4)	5 (6.2)	0.277
*SMC1A*	5 (6.1)	2 (2.5)	0.443
*SMC3*	3 (3.7)	4 (4.9)	0.720
*STAG2*	3 (3.7)	3 (3.7)	1.000
*MT-CYB*	1 (1.2)	4 (4.9)	0.210
*PHF6*	3 (3.7)	2 (2.5)	1.000
Highly expressed gene, no. (%)			
High *ERG*	38 (46.3)	43 (53.1)	0.389
High *BAALC*	41 (50.0)	40 (49.4)	0.937
High *MN1*	40 (48.8)	41 (50.6)	0.815
High *WT1*	37 (45.1)	44 (54.3)	0.240
High *LEF1*	39 (47.6)	42 (51.9)	0.584
High *DNMT3A*	43 (52.4)	38 (46.9)	0.481
High *DNMT3B*	29 (35.4)	52 (64.2)	<0.001
High *ITPR2*	44 (53.7)	37 (45.7)	0.308
High *ATP1B1*	40 (48.8)	41 (50.6)	0.815
High *TCF4*	27 (32.9)	54 (66.7)	<0.001
High *MECOM*	43 (52.4)	38 (46.9)	0.481
High *GATA2*	31 (37.8)	50 (61.7)	0.002
High *P2RY14*	32 (39.0)	49 (60.5)	0.006
High *INSR*	53 (64.6)	28 (34.6)	<0.001
High *TET1*	31 (37.8)	50 (61.7)	0.002
High *TET2*	43 (52.4)	38 (46.9)	0.481
High *ITGA3*	29 (35.4)	52 (64.2)	<0.001
Normal karyotype, no. (%)	43 (53.1)	36 (44.4)	0.346
Complex karyotype, no. (%)	4 (4.9)	19 (23.5)	<0.001
Treatment induction			
Transplantation, no. (%)	46 (56.1)	31 (38.3)	0.028
Risk status based on NCCN guidelines, no. (%)			
Favorable risk	32 (39.0)	12 (14.8)	<0.001
Intermediate risk	33 (40.2)	21 (25.9)	0.051
Poor risk	17 (20.7)	48 (59.3)	<0.001

### Patients with AML having higher prognostic scores in the training set demonstrated poorer prognosis

**Figure 2:**
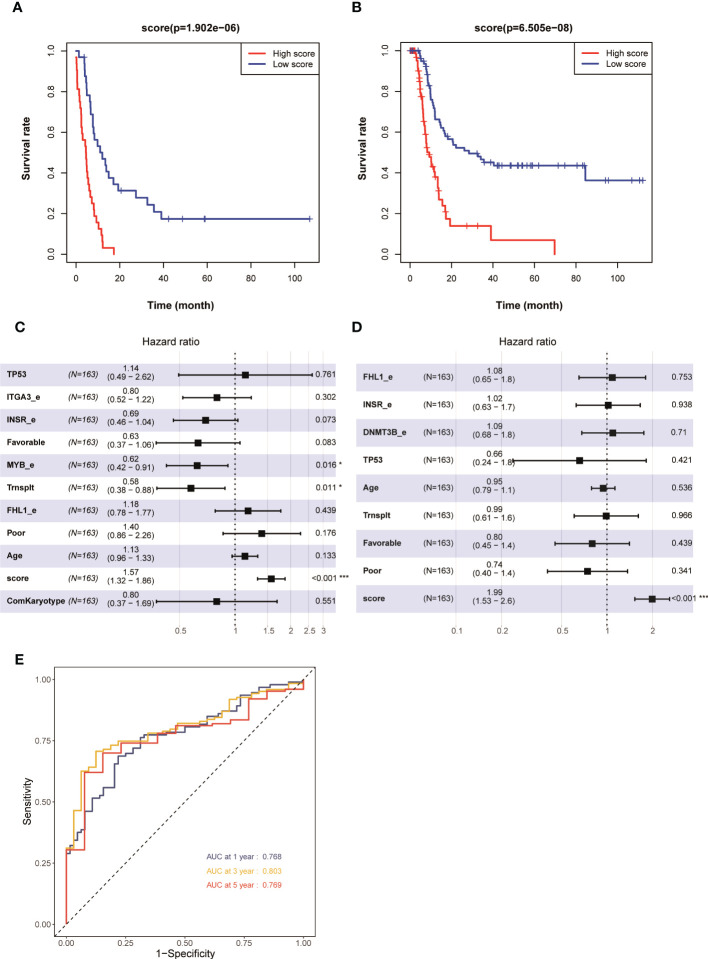
Kaplan–Meier survival curves of patients with AML having high and low risk scores in 163 patients with AML (none-m3) in the TCGA database: **(A)** OS and **(B)** EFS. Multivariate analyses of clinical outcomes for patients with AML. **(C)** OS and **(D)** EFS. **(E)** ROC curves for predicting 1-, 3-, and 5-year survival in the training set. *p < 0.05, ***p < 0.001.

The risk scores of each patient with AML in the training set in the TCGA database were obtained using the risk score calculation formula. The patients were divided into a high-risk group (*N* = 81) and a low-risk group (*N* = 82) using the median value of the risk score as the cutoff value. The overall survival (OS) (*P* < 0.001) and even-free survival (EFS) (*P* < 0.001) in the low-risk group were significantly higher than those in the high-risk group (**Figures 2A, B**). The results of the multivariate Cox regression analysis showed that the risk score, transplantation, and high expression of MYB could be used as independent influencing factors for OS in patients with AML (**Figure 2C**). Also, the risk score and transplantation could be used as independent influencing factors for EFS in patients with AML (**Figure 2D**). The AUCs for 1, 3, and 5 years in the training set were all greater than 0.750 (**Figure 2E**). The aforementioned results showed that patients with AML having higher prognostic scores in the training set had a poorer prognosis, and the model had a higher predictive value for the prognosis of AML in the training set.

### Patients with AML having higher prognostic scores in the validation set GSE6891 had a poorer prognosis

**Figure 3:**
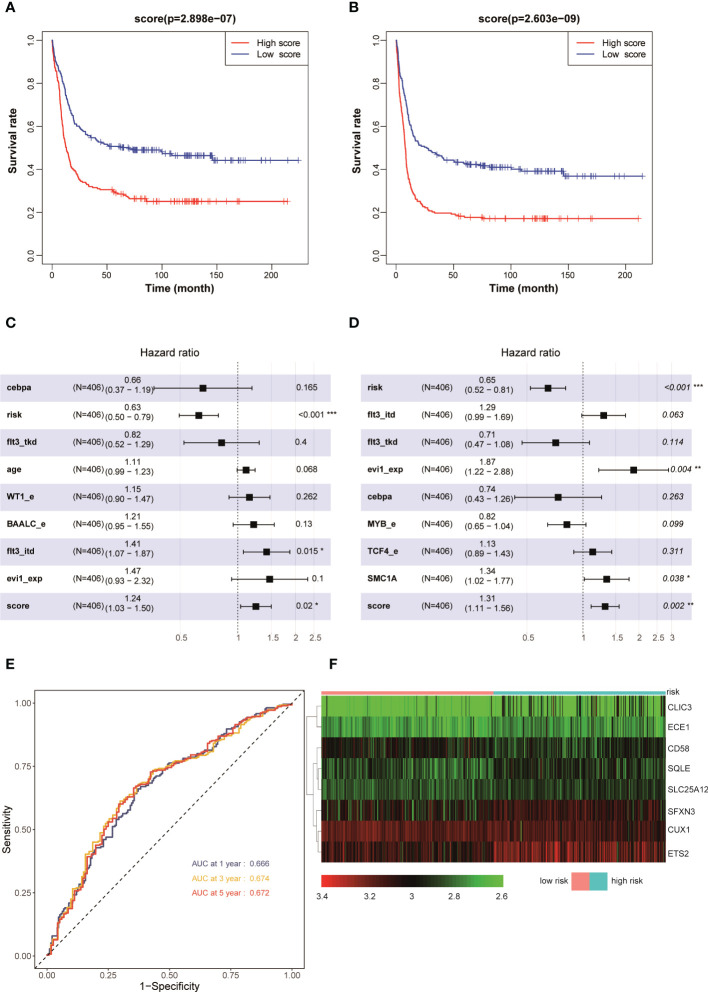
Kaplan–Meier survival curves of AML patients with high and low risk scores in GSE6891: **(A)** OS and **(B)** EFS. Multivariate analyses of clinical outcomes for patients with AML in GSE 6891: **(C)** OS and **(D)** EFS. **(E)** ROC curves for predicting 1-, 3-, and 5-year survival in the validation set. **(F)** Heat map of the eight scoring model genes in GSE6891. *p < 0.05, **p < 0.01, ***p < 0.001.

The aforementioned results demonstrated that the AML prognostic scoring model had a high predictive value in the training set. The evaluation on the prognostic scoring system was performed again in the GSE6891 validation set to further evaluate the performance of the prognostic scoring model. The risk score of each patient with AML in the validation set was obtained using the risk score calculation formula. Based on the median value of the risk score as the cutoff value, the patients were divided into a high-risk group (*N* = 203) and a low-risk group (*N* = 203). The results showed that the OS (*P* < 0.001) and EFS (*P* < 0.001) in the low-risk group were significantly higher than those in the high-risk group (**Figures 3A, B**). The results of the multivariate Cox regression analysis showed that the risk score, *FLT3-ITD*, and NCCN risk stratification could be used as independent influencing factors for OS in patients with AML (**Figure 3C**). Also, the risk score, NCCN risk stratification, high expression of *EVI1*, and *SMC1A* mutation could be used as independent influencing factors for EFS in patients with AML (**Figure 3D**). The AUCs for 1, 3, and 5 years in the training set were all greater than 0.650 (**Figure 3E**). The expression levels of *CUX1* and *CD58* in the eight modeling genes were higher, while the expression levels of other genes were lower, in the low-risk group compared with the high-risk group (**Figure 3F**). The aforementioned results showed that the model had a high predictive value for the prognosis of AML in the validation set.

### Prognostic scoring model was used for patients with AML having intermediate risk and patients with normal karyotype: patients with higher scores had a poorer prognosis

**Figure 4:**
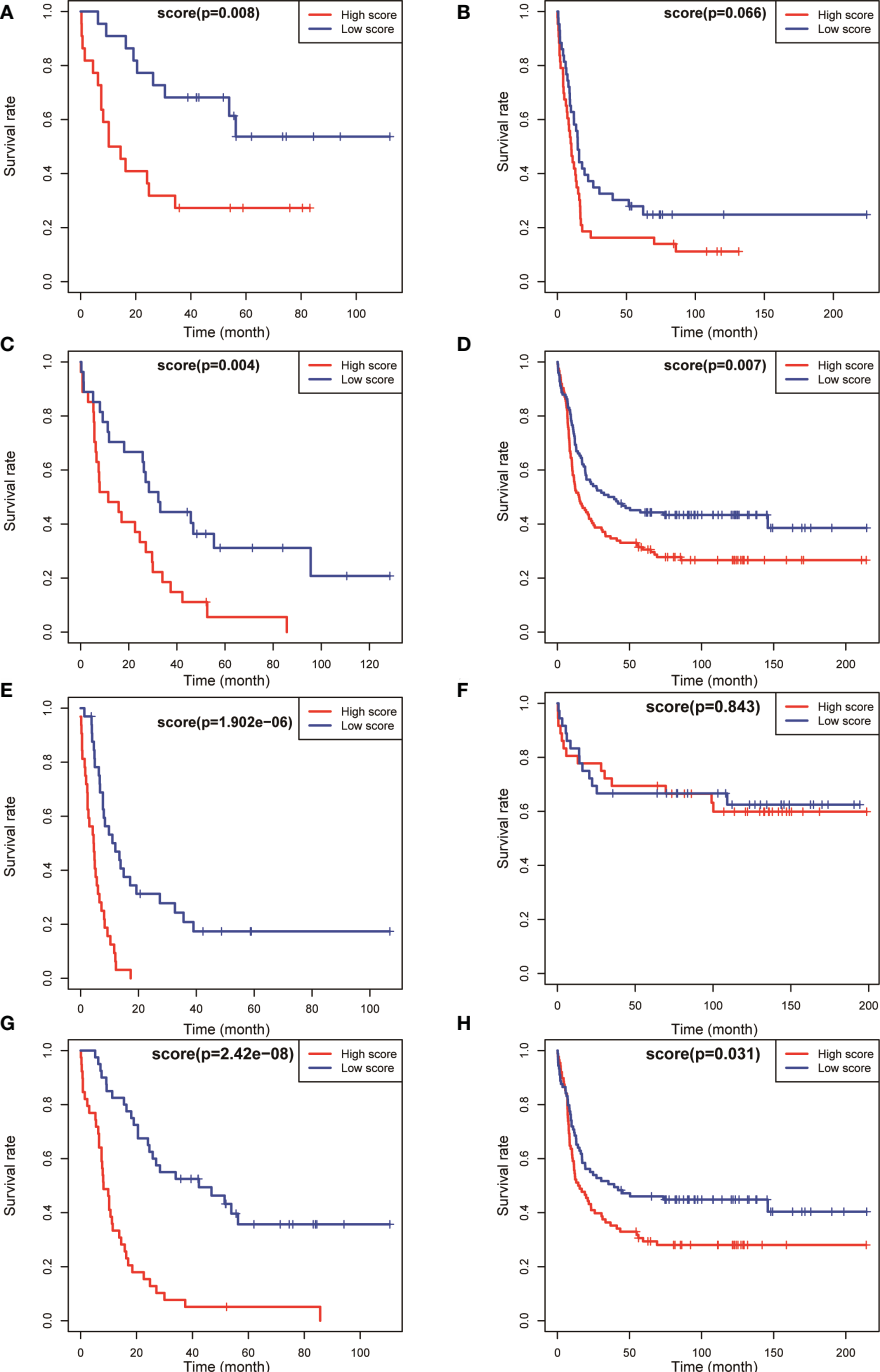
Kaplan–Meier survival analysis of OS for patients in different NCCN risk strata was performed based on high and low risk scores. OS of the favorable-risk subgroup in TCGA **(A)** and GSE6891 **(B)**; OS of the intermediate-risk subgroup in TCGA **(C)** and GSE6891 **(D)**; OS of the poor-risk subgroup in TCGA **(E)** and GSE6891 **(F)**; and OS of the normal-karyotype subgroup in TCGA **(G)** and GSE6891 **(H)**.

**Figure 5:**
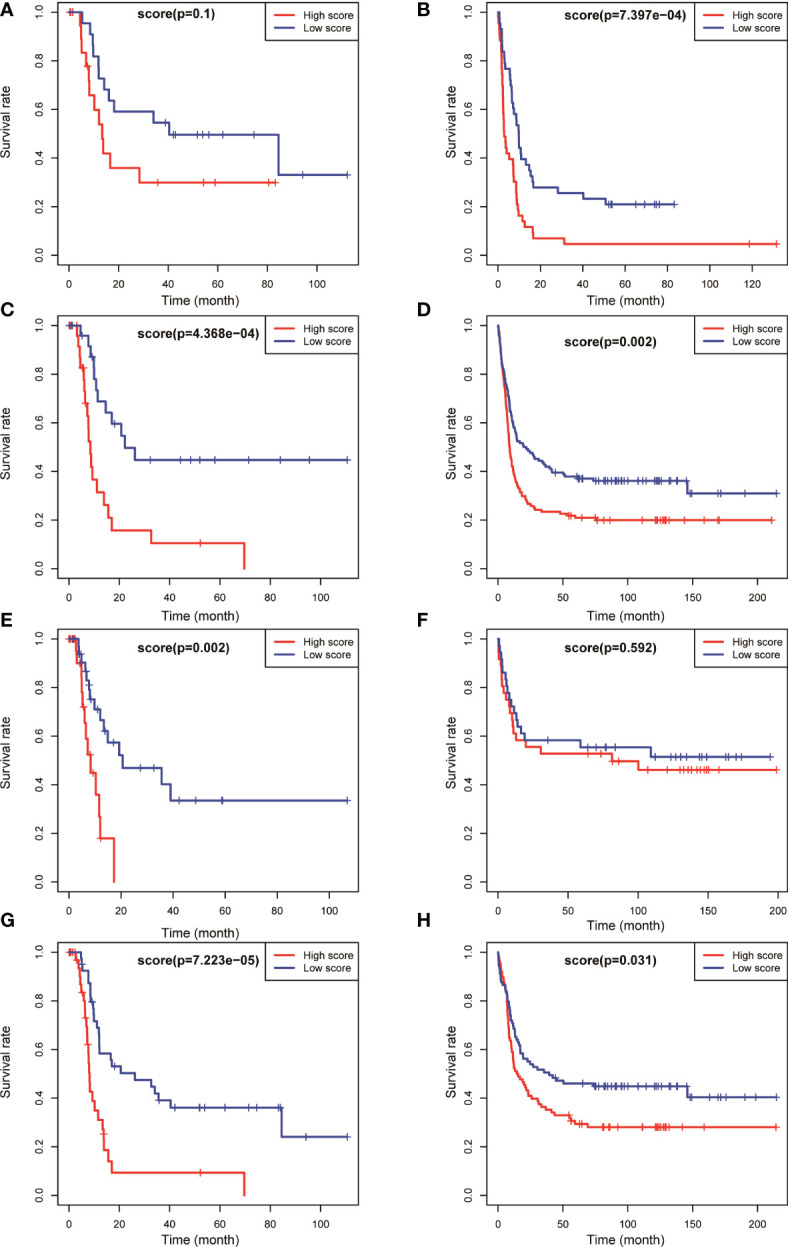
Kaplan–Meier survival analysis of EFS for patients in different NCCN risk strata was performed based on high and low risk scores. EFS of the favorable-risk subgroup in TCGA **(A)** and GSE6891 **(B)**; EFS of the intermediate-risk subgroup in TCGA **(C)** and GSE6891 **(D)**; EFS of the poor-risk subgroup in TCGA **(E)** and GSE6891 **(F)**; and EFS of the normal-karyotype subgroup in TCGA **(G)** and GSE6891 **(H)**.

The aforementioned results confirmed that the model had a higher predictive value for the overall prognosis of patients with AML. The OS and EFS of patients with AML in different risk stratification subgroups in the training and validation sets were analysed to validate the predictive value of the model for the prognosis of patients with AML in NCCN risk stratification subgroups. According to the prognostic risk, 163 patients with AML in training set TCGA were divided into 3 groups: low-risk group (44 cases), intermediate-risk group (54 cases), and high-risk group (65 cases). Using the median risk score as the cutoff value, patients were divided into high-score group and low-score group in each risk subgroup. The results showed that patients with AML having high prognostic scores had poorer OS in the low-risk (*P* = 0.008, **Figure 4A**), intermediate-risk (*P* = 0.004, **Figure 4C**), and high-risk (*P* < 0.001, **Figure 4E**) prognostic stratification subgroups of the training set TCGA. Similarly, according to the prognostic risk, 406 patients with AML in the validation set GSE6891 were divided into 3 groups: low-risk group (*N* = 86), intermediate-risk group (*N* = 248), and high-risk group (*N* = 72). Patients with AML having high prognostic scores had poorer EFS in the intermediate-risk (*P* = 0.007, **Figure 4D**) prognostic stratification subgroup of the validation set GSE6891. Further analysis of the EFS of patients with AML in different risk stratifications showed that these patients with high prognostic scores had poorer EFS in the intermediate-risk (*P* < 0.001, **Figure 5C**) and high-risk (*P* = 0.002, **Figure 5E**) subgroups of the training set TCGA. Similarly, patients with AML having high prognostic scores had poorer EFS in the low-risk (*P* < 0.001, **Figure 5B**) and intermediate-risk (*P* = 0.002, **Figure 5D**) subgroups of the validation set GSE6891.

We selected patients with AML having normal karyotypes in the training and validation sets (*N* = 79 in TCGA, *N* = 177 in GSE6891) and divided the patients with normal karyotypes into high- and low-score groups using a median value of risk score as a cutoff value to explore whether this risk model had a predictive effect in patients with AML having normal karyotypes. The OS and EFS of patients were analysed. The results showed that patients with normal karyotypes and high scores had poorer OS (*P* < 0.001, **Figure 4G**; *P* = 0.031, **Figure 4H**) in the training and validation sets. Also, patients with AML having high scores had poorer EFS than those with low scores in the training and validation sets (*P* < 0.001, **Figure 5G**; *P* = 0.031, **Figure 5H**).

The aforementioned results indicated that the prognostic model could be used to effectively predict the prognosis of patients with AML in the NCCN prognostic risk stratification subgroup, especially in the group with NCCN moderate prognosis. The analysis also showed that the model had a high predictive value for the prognostic risk in AML populations with normal karyotype (**Figures 4**, **5**).

### Proposed scoring system quantified the risk in a manner consistent with NCCN risk stratification

**Figure 6:**
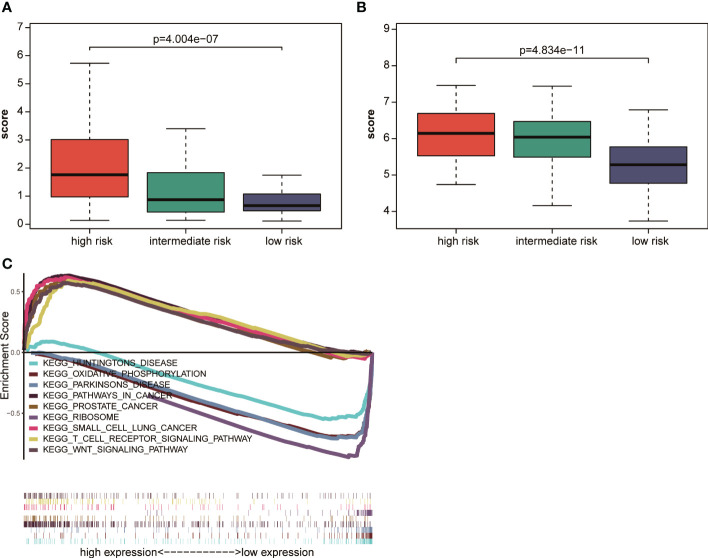
Correlation analysis of NCCN clinical risk stratification and prognostic scores **(A)** in TCGA and **(B)** in GSE6891. **(C)** GSEA analysis of cancer hallmark signaling pathway or biological process between the high-risk and low-risk groups in the TCGA dataset.

According to NCCN guidelines on risk stratification, patients with AML were divided into low-risk group, intermediate-risk group, and high-risk group. Patients with AML having high prognostic scores had a poor prognosis. In this study, the correlation between the AML prognostic scores and clinical risk stratification was explored. The results showed that in the TCGA database, the prognostic score of AML decreased with the decrease in NCCN risk (**Figure 6A**), and the prognostic score measured using this model for patients with high-risk NCCN prognosis increased significantly. Similar results were observed in GSE6891 (**Figure 6B**). The aforementioned results indicated that the AML prognostic scoring system had a significant correlation with NCCN clinical risk stratification, and could be used as another accurate way to assess patients’ prognostic risk.

### Tumor-associated pathway activation within the group of patients with high-risk scores

Patients were divided into a high-risk group and a low-risk group using the median prognostic score as a cutoff value in TCGA to explore the pathways related to the high- and low-risk groups, and then GSEA pathway enrichment analysis was performed. The results showed that the small cell lung cancer signaling pathway, Wnt signaling pathway, and prostate cancer signaling pathway were significantly enriched in the high–prognostic score group. Huntington’s signaling pathway, Parkinson’s disease signaling pathway, and oxidative phosphorylation signaling pathway were significantly enriched in the low–prognostic score group (**Figure 6C**). The results showed significant difference in the enrichment of related signaling pathways or biological processes between the high-risk and low-risk groups. Also, the tumor-related characteristic pathways were significantly activated in patients with high prognostic scores.

## Discussion

Recently, a large number of molecular markers related to the onset and prognosis of AML, such as fusion genes *CBFB–MYH11*, *RUNX1–RUNX1T1*, and *BCR-ABL1* caused by chromosome aberrations and mutant genes *NPM1*, *TP53*, and *ASXL1*, have been gradually discovered and investigated. The risk-stratified therapies have saved the lives of tens of thousands of patients with AML. More than half of the patients cannot survive for more than 5 years owing to the heterogeneity of AML despite the development of AML risk stratification (7). AML prognostic stratification needs to be more precise so that as many patients as possible receive the treatment that is most appropriate for them (8). This study demonstrated that the risk stratification based on the eight-gene risk scoring system was consistent with the NCCN guidelines. Patients with high NCCN risk had higher prognostic scores. For patients with intermediate-risk stratification of NCCN or CN-AML, those with high scores had a worse prognosis than those with low scores. The results of this study strongly suggested that the proposed risk prediction model could be used to further quantify the prognostic risk of patients with NCCN intermediate risk and those with normal karyotype. Accurate risk stratification might lay the foundation for accurate leukemia treatment.

In this study, we established a risk prediction model containing eight genes (*CD58, CLIC3, CUX1, ECE1, ETS2, SFXN3, SLC25A12*, and *SQLE*). The *CD58* encoded protein is a ligand for the CD2 protein and has the function of adhering and activating T lymphocytes (9). *C*hloride intracellular channel 3 (*CLIC3*) is a member of the p64 family and is located in the nucleus. This protein may be involved in cell growth control. Studies have shown that *CLIC3* promotes cancer progression through its glutathione-dependent oxidoreductase activity (10). The *CUX1*-encoded protein regulates gene expression and plays a role in cell cycle progression. *CUX1* is a haplotype tumor suppressor gene on chromosome 7 and is often inactivated in AML (11). High expression of *ETS2* indicates a poor prognosis of AML (12). *SLC25A12* encodes a calcium-bound mitochondrial carrier protein involved in the exchange of aspartic acid and glutamic acid across the inner mitochondrial membrane (13). *MYC* promotes cholesterol biosynthesis in tumor cells and supports cell proliferation through *SQLE* (14). The aforementioned genes are related to tumor development and are differentially expressed in patients with AML and healthy people.

AML with a normal karyotype is stratified as intermediate risk according to the NCCN guideline, but the clinical outcome of this group of patients is uneven. The AML risk classification is difficult for approximately half of patients with CN-AML (15). The molecular heterogeneity of CN-AML is not fully reflected in the current classification systems (16). The aforementioned group of patients with AML is challenging to stratify, and hence more precise stratification methods and further molecular mutations are required (17). In recent years, multiple prognostic markers have been used to identify patient groupings with different prognoses. For example, patients with low ERG, low EVI1, and high PRAME levels had a positive prognosis (16). The availability of full AML genome sequence information might lead to the discovery of additional prognostic indicators in patients with AML. As a result, new methodologies are required in which a large amount of prognostic data is combined to allow precise outcome prediction for specific treatments (18). It is necessary to maximize clinical outcome prediction by combining a larger number of known biomarkers, rather than employing an individual or small groups of biomarkers. The proposed scoring system precisely quantified the clinical risk of these patients, and updated and supplemented the content of the existing prognostic classification guidelines. The present study demonstrated that this AML prognostic scoring model was designed to improve the prognostic risk stratification of patients with AML. However, this study had some limitations. The number of cases was relatively small, and hence a large sample size is required to validate the prognostic risk model. In addition, more patients from multiple centers are required to make the patient cohort more representative.

## Conclusions

In summary, this study was novel in reporting the DEG-based prognostic risk model. It demonstrated that a high prognostic score was an independent risk factor for the prognosis of patients with AML. It can be used to update the existing NCCN guidelines on prognostic risk stratification. Also, it might provide an important basis for the precise treatment of patients with AML.

## Methods

### Acquisition and collation of AML-related data

Data were available in public data sets, including (1) The Cancer Genome Atlas (TCGA) database and (2) Gene Expression Omnibus (GEO) database. The gene expression data on bone marrow and peripheral blood for 163 patients with non-M3 AML and corresponding patient information (including the patients’ age, sex, clinical characteristics, fusion gene, mutation, survival status, survival time, and so on) were downloaded from the TCGA database (https://portal.gdc.cancer.gov), and the gene expression data were standardized as a training set for subsequent investigation. The gene expression data of GSE6891, GSE9476, and GSE1159 patients and the corresponding clinical information (including patients’ age, sex, survival status, survival time, and so on) were downloaded from the GEO database (https://www.ncbi.nlm.nih.gov/geo/). The raw data were processed using the robust multi-array averaging function of the R package. The AML patients’ specimens and healthy human bone marrow or peripheral blood samples were included from GSE9476 (*n* = 46) and GSE1159 (*n* = 290), which were used for differential gene analysis; the GSE6891 (*n* = 406) database contained the clinical information required for model research part, as a model validation set for subsequent studies.

### Screening of DEGs

The DEGs in the microarray data set were screened using the R language limma package. The difference in gene expression was expressed by the logarithm (log2FC) of the *P* value and the fold change. The genes with adj.*P <*0.05 and |log2FC| >1 were regarded as DEGs. Subsequently, the DEGs in the GSE9476 and GSE1159 data sets were screened.

### Construction of the prognostic risk scoring model

A secondary screening was performed for DEGs obtained by the aforementioned screening to obtain DEGs shared by the GSE9476 and GSE1159 data sets. The survival package of R language was used to perform univariate Cox regression analysis to screen the genes related to poor prognosis [hazard ratio (HR)>1, *P <*0.05] and use them as candidate genes for constructing the model. A prognostic risk scoring model was constructed by multivariate Cox regression analysis. The risk score was calculated using the following formula: risk score = expression level of gene 1 × multivariate Cox regression coefficient 1 +… + expression level of gene N × multivariate Cox regression coefficient *N* (where *N* represented the number of genes). The risk score of each patient was calculated, and the patients were divided into high-risk and low-risk groups using the median as a cutoff value.

### Performance evaluation of the prognostic risk scoring model

The risk score of each patient in the training set was calculated using the aforementioned formula. Based on the result, the patients were divided into high-risk and low-risk groups. The receiver operating characteristic (ROC) curve was plotted using the R language survival ROC package, and the areas under the curve (AUC) of patients after 1, 3, and 5 years in the training set were calculated. The Kaplan–Meier (KM) survival curve was plotted using the R language survminer package. Moreover, based on clinical information such as age, sex, and tumor stage of patients with AML, a multivariate Cox regression model was used to analyse whether the risk score was an independent factor in judging the poor prognosis of AML. The prediction performance of the model in the training and validation sets was evaluated according to the AUC value and the difference in the KM survival rate between the groups.

### Correlation analysis between prognostic score and clinical risk

The risk score of each patient in the training set was calculated using the aforementioned formula. Based on the result, the patients were divided into high-risk and low-risk groups. According to the NCCN risk stratification of patients, the Wilcoxon test was performed using R language to conduct a correlation analysis between the prognostic score and clinical risk.

### Gene set enrichment analysis for the risk scoring model in AML

All genes from the RNA-seq of the TCGA AML cohort were pre-ranked using the risk score with the median value as the cutoff. Gene set enrichment analysis (GSEA) v.4.0 was used for the analysis. The Kolmogorov–Smirnov statistic was used to obtain enrichment scores, which were then normalized to account for the size of each gene set.

### Statistical analysis

Statistical analysis was performed using software R (version 3.6.2) for all data. A *P* value <0.05 indicated a statistically significant difference.

## Data availability statement

The gene expression data on bone marrow and peripheral blood for 163 patients were downloaded from the TCGA database (https://portal.gdc.cancer.gov), and the gene expression data were standardized as a training set for subsequent investigation. The gene expression data of GSE6891, GSE9476, and GSE1159 patients and the corresponding clinical information were downloaded from the GEO database (https://www.ncbi.nlm.nih.gov/geo/).

## Ethics statement

The studies involving human participants were reviewed and approved by Medical Ethics Committee of the Fifth Medical Center of the Chinese People’s Liberation Army General Hospital.

## Author contributions

(I) Conception and design: FL. (II) Administrative support: FL, B-LH. (III) Provision of study materials or patients: FL, HW. (IV) Collection and assembly of data: J-JY, SF, Y-NW, KQ, Y-FJ, NL, R-QS, W-JG. (V) Data analysis and interpretation: YY, HW. (VI) Manuscript writing: all authors. (VII) Final approval of manuscript: all authors. All authors contributed to the article and approved the submitted version.
