# Supplementary material for: A novel scoring system for the quantitative prediction of prognosis in acute myeloid leukemia
Source: Front Oncol. 2023 Mar 30;13:1144403. doi: 10.3389/fonc.2023.1144403 (PMC10098320; doi:10.3389/fonc.2023.1144403)
Supplement: Supplementary file 1 [file Table_1.docx]

Table S1. UniCox analysis of DEGs（P＜0.05）

| Gene ID | HR（Hazard Ratio） | P value |
| --- | --- | --- |
| ETS2 | 1.928123122 | 2.68E-06 |
| AK1 | 1.742571428 | 8.58E-06 |
| LDLRAP1 | 1.711987774 | 0.000103693 |
| ECE1 | 1.379012743 | 0.000118864 |
| CLIC3 | 1.533550835 | 0.000133985 |
| GZMB | 1.295218681 | 0.000268076 |
| SH3BP5 | 1.427456131 | 0.000352752 |
| CUX1 | 0.440223464 | 0.000499365 |
| PPP1R16B | 1.311362445 | 0.000980767 |
| SQLE | 1.507469333 | 0.001068624 |
| RUNX3 | 1.286456394 | 0.001071642 |
| SFXN3 | 1.42724354 | 0.00134426 |
| GZMH | 1.281770252 | 0.001385478 |
| LSP1 | 1.243768952 | 0.001464891 |
| CLEC11A | 0.879998244 | 0.001478842 |
| STAT4 | 1.354538819 | 0.001591713 |
| CD22 | 1.285893241 | 0.002346554 |
| SLC25A12 | 2.365188804 | 0.002873102 |
| SLC2A5 | 1.256140073 | 0.002889163 |
| MTMR1 | 1.901359914 | 0.002960226 |
| CD58 | 0.670387576 | 0.003532485 |
| GCLM | 1.350278292 | 0.003941118 |
| ALDH1A1 | 1.15134629 | 0.004295781 |
| FHL2 | 1.330864464 | 0.004633802 |
| GAS2L1 | 1.294984207 | 0.008327768 |
| C7orf23 | 0.648057712 | 0.00862172 |
| BIN1 | 1.220039188 | 0.008704581 |
| SLC22A4 | 0.735447709 | 0.01079914 |
| KIF21B | 1.413354002 | 0.011475188 |
| GNLY | 1.170472798 | 0.012349321 |
| CASP2 | 0.652688769 | 0.013821458 |
| SPOCK2 | 1.219401065 | 0.014017909 |
| ARL4C | 1.255809124 | 0.014523117 |
| MYBL1 | 1.38462984 | 0.014799313 |
| LGALS3BP | 1.107312475 | 0.015914775 |
| PFKP | 1.375537097 | 0.015993095 |
| EVL | 1.26368575 | 0.017441253 |
| KLRD1 | 1.259424486 | 0.019118702 |
| MMD | 1.423511182 | 0.019339208 |
| CDC25B | 1.485838742 | 0.019442132 |
| HOXA10 | 1.122418659 | 0.020089774 |
| ISG20 | 1.273163998 | 0.020217383 |
| ZNF334 | 1.175481104 | 0.021926333 |
| PRF1 | 1.18968737 | 0.02245235 |
| MAP7 | 1.180051689 | 0.022973702 |
| NR1H3 | 1.314159014 | 0.023678604 |
| FLT3LG | 1.296143014 | 0.023809675 |
| THBS1 | 1.097059717 | 0.024740075 |
| IVNS1ABP | 0.696017516 | 0.024915832 |
| ITGB3 | 1.118035508 | 0.025540673 |
| TPM1 | 1.217224127 | 0.02657721 |
| TGFBR3 | 1.308469926 | 0.028743933 |
| HOMER3 | 0.811842853 | 0.028985717 |
| LTB | 1.205320245 | 0.029057183 |
| DAPK1 | 1.192634992 | 0.029298842 |
| PPBP | 1.08554529 | 0.029513528 |
| MEIS1 | 1.114578419 | 0.030178254 |
| PFKM | 1.268438089 | 0.031093125 |
| PF4 | 1.092256453 | 0.031890751 |
| GZMM | 1.288322204 | 0.033048943 |
| ALDH2 | 1.122888707 | 0.033740285 |
| EZR | 1.375312542 | 0.035443978 |
| HOXB5 | 1.115878005 | 0.035492949 |
| CLIP2 | 1.16648372 | 0.037086638 |
| RBM38 | 1.200323994 | 0.038577745 |
| CPT1A | 1.458247362 | 0.039805099 |
| PBX3 | 1.129143832 | 0.042440938 |
| TBX21 | 1.230942458 | 0.04418074 |
| PRKCH | 1.190977458 | 0.044360248 |
| HOXA5 | 1.080376562 | 0.046364409 |
| RABEP2 | 1.351119729 | 0.04778358 |
| HOXB6 | 1.109242375 | 0.048819632 |
| ABCC1 | 1.275713266 | 0.049288051 |
| ENO2 | 1.179271389 | 0.049713166 |
